# Improving Glycerol Photoreforming Hydrogen Production Over Ag_2_O-TiO_2_ Catalysts by Enhanced Colloidal Dispersion Stability

**DOI:** 10.3389/fchem.2020.00342

**Published:** 2020-05-19

**Authors:** Zhi Yang, Weilin Zhong, Ying Chen, Chao Wang, Songping Mo, Jingtao Zhang, Riyang Shu, Qingbin Song

**Affiliations:** ^1^Guangdong Provincial Key Laboratory on Functional Soft Condensed Matter, School of Materials and Energy, Guangdong University of Technology, Guangzhou, China; ^2^Macau Environmental Research Institute, Macau University of Science and Technology, Macao, China

**Keywords:** hydrogen production, bio-glycerol, photoreforming, colloidal dispersion stability, Ag_2_O-TiO_2_ nanoparticles

## Abstract

Solar-driven photocatalytic reforming of biomass-derived resources for hydrogen production offers a sustainable route toward the generation of clean and renewable fuels. However, the dispersion stability of the catalyst particles in the aqueous phase hinders the efficiency of hydrogen production. In this work, a novel method of mixing Ag_2_O-TiO_2_ photocatalysts with different morphologies was implemented to promote colloidal dispersion stability, thereby improving hydrogen production performance. A series of Ag_2_O-TiO_2_ nanoparticles with different morphologies were synthesized, and their dispersion stabilities in aqueous phase were investigated individually. Two types of Ag_2_O-TiO_2_ particles with different morphologies under certain proportions were mixed and suspended in glycerol aqueous solution without adding any dispersant for enhancing dispersion stability while reacting. From the results, photocatalytic hydrogen production was found to be strongly correlated to colloidal dispersion stability. The mixed suspension of Ag_2_O-TiO_2_ nanosphere and nanoplate achieved an excellent colloidal dispersion stability without employing any additives or external energy input, and the photoreforming hydrogen production obtained from this binary component system was around 1.1–2.3 times higher than that of the single-component system. From the calculated hydrogen production rate constants between continuous stirring and the binary system, there was only <6% difference, suggesting an efficient mass transfer of the binary system for photoreforming hydrogen production. The proposed method could provide some inspiration to a more energy-efficient heterogeneous catalytic hydrogen production process.

## Introduction

Photoreforming hydrogen production route has been attracting great attention due to its integration of both solar energy and renewable sources utilization (Liu et al., 2014; Yu et al., 2015; Sadanandam et al., 2017). With the presence of renewable sacrificial organic compounds [e.g., glycerol (Shen Y. et al., 2019), lactic acid (Fu et al., 2019), or wood (Kawai and Sakata, 1980)],

the reaction efficiency of H_2_ generation could be significantly improved as those compounds are more readily to combine with photo-generated h^+^ than water splitting. Actually, the redox reaction between water and organic compounds into a one-step process could be defined as photoreforming which is a valid approach to produce H_2_ as it is more thermodynamically feasible than pure water splitting (Fu et al., 2008). It is worth noting that a large number of biomass-derived substrates, such as bio-alcohols, could be used for this photoreforming hydrogen production process. Among those biomass-derived substrates, glycerol (C_3_H_8_O_3_) as a by-product of biodiesel production attracts special interest for hydrogen production for its low cost and excess production (Daskalaki et al., 2011; Gombac and Falqui, 2016). In our recent studies, glycerol has been found to have great potential for both efficient thermo-chemical and photo-chemical hydrogen production (Wang et al., 2015, 2017a,b; Ni et al., 2017).

**Graphical Abstract:**
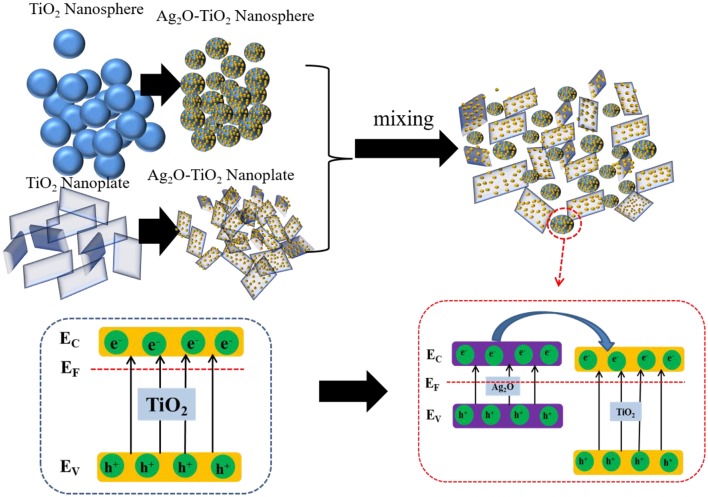
Hydrogen production by enhanced colloidal dispersion stability.

Titanium dioxide, one of the most promising photocatalysts, has been widely studied for photoreforming hydrogen production (Petala et al., 2015). However, there are some obstacles for further practical applications of bare TiO_2_: the severe electron-hole recombination of bare TiO_2_ catalyst caused by a mismatch between photo-excited charge carriers life span and redox reaction slow kinetics, and this could lead to low energy conversion efficiency (Patrocinio et al., 2015; Litke et al., 2017); the spontaneous aggregation of TiO_2_-based particles when they are being suspended in aqueous phase due to their exposed high-surface energy facets for particular crystals (Chen et al., 2015; Zhang et al., 2015). Thus, it is desirable to maintain certain dispersion stability during reaction and suppress electron-hole recombination to better achieve hydrogen production. Efforts in previous investigations have been made to enhance the TiO_2_-basis photocatalytic activity (Yang et al., 2013; Pan et al., 2018; Shen J. et al., 2019; Wang W. et al., 2019). In our previous studies, it was found that photoreforming H_2_ production could be improved by coupling other metal oxide semiconductors to bare TiO_2_ with a sol–gel method (Wang et al., 2017a). In particular, an efficient catalytic hydrogen production was achieved over Ag_2_O-TiO_2_ catalyst. This was mainly because Ag_2_O composite could form hetero-structures with TiO_2_, which could efficiently provide the rapid separation sites for the photo-generated electrons and holes.

Photocatalytic efficiency in an aqueous phase environment is found to be influenced by the catalyst aggregation to some extent (Li et al., 2010). In the works of Lakshminarasimhan et al. (2008), they concluded that the higher photocatalytic hydrogen yield was effected by the particle agglomeration of TiO_2_. Besides, physical dispersion such as ultrasonic dispersion and mechanical dispersion and chemical dispersion such as dispersant or surfactant addition and nanoparticle surface modification were pointed out to be effective for improving the stability of TiO_2_ particles in water (Kim and Nishimura, 2012; Othman et al., 2012). However, those methods requiring extra external energy input (e.g., ultrasonic dispersion, mechanical stirring, or electromagnetic stirring, etc.) obviously break the energy balance and increase the cost of large-scale application of photocatalytic hydrogen production. In other previous studies, it was found that the particle agglomeration could be minimized by controlling the pH of the suspension (Zhang et al., 2018), applying silane coupling agent modification of TiO_2_ (Wang C. et al., 2019) or fluorinating TiO_2_ particles by fluorine gas etc. (Kim et al., 2012). Kim et al. (2012) reported that the photocatalytic activity would be improved due to the surface fluorination of titanium dioxide by enhancing the dispersion stability of the TiO_2_ in the organic reagents (Kim and Nishimura, 2012). Theoretically, photocatalytic activity is greatly affected by the dispersion stability which is directly influenced by the electrostatic interactions between the solid surface and generated ions. However, the approaches of TiO_2_ surface modification may sacrifice the surface-active sites, resulting in a lower surface catalytic reaction efficiency. Those methods of regulating the composition of the liquid substrates (such as adjusting pH value) may also increase the complexity of the aqueous-phase reaction system and interfere with the mass transfer of the reactions. In addition, the high cost of surfactants and the disposal of generated residues would also be derivative issues. In our previous studies, it was discovered that TiO_2_-H_2_O nanofluids could be stabilized through the addition of ultra-thin ZrP nanoplatelets (Liu et al., 2015b). The means of mixing TiO_2_ particles with different shapes may be beneficial to the dispersion stability (Shao et al., 2015). We have achieved a preliminary enhancing hydrogen production by mixing two types of bare TiO_2_ with different shapes (Shao et al., 2015). The mixed suspension of TiO_2_ nanosphere and nanosheet still showed great colloidal dispersion stability and photocatalytic hydrogen production promoting at a specific mixing ratio.

Herein, this study attempts to investigate the dispersion stability of Ag_2_O-TiO_2_-based photocatalysts with different morphologies and its effect on photocatalytic activity for photoreforming hydrogen production. Various Ag_2_O-TiO_2_ nanoparticles with different morphologies were synthesized, and their microstructures were detected by X-ray diffraction (XRD), Brunauer-Emmett-Teller measurements (BET), and high-resolution transmission electron microscopy (HRTEM) analysis etc. The dispersion stabilities of the aqueous suspensions were characterized using zeta potential measurements and a Turbiscan Stability method. Based on the obtained results of previous studies, binary Ag_2_O-TiO_2_ systems were introduced by dispersing a certain ratio of two types of Ag_2_O-TiO_2_ nanoparticles to enhance the dispersion stabilities. Hydrogen production from photoreforming of glycerol aqueous solution was carried out to examine the relationship between dispersion stability and photocatalytic activity.

## Experiment

### Synthesis of Various Shapes of Ag_2_O-TiO_2_ Nanoparticles

The reason why Ag_2_O was utilized in the experiment is that Ag_2_O-TiO_2_ has the strongest photocatalysis ability among ZnO_2_-TiO_2_, Bi_2_O_3_-TiO_2_, and Ag_2_O-TiO_2_ due to its narrow band gap and absorption of more spectra energy (Wang et al., 2017a) and the high self-stable shown in the Ag_2_O to eliminate the external influence to the system stability (Yu et al., 2016).

All chemicals were purchased from Sigma-Aldrich Trading Co. Ltd., and the reagents with analytical grade were used as received without further purification. The deionized water was prepared by Millipore Milli-Q ultrapure water purification systems with a resistivity larger than 18.2 MΩ. Synthesis of various shapes of Ag_2_O-TiO_2_ nanoparticles could be summarized as follows: synthesis of TiO_2_ with various shapes and compounding Ag_2_O with the prepared TiO_2_. According to the previously study, TiO_2_ could be prepared to provide the desired morphologies by several methods. TiO_2_ nanosphere with the average diameter of 32 nm was directly used as received. The as-prepared TiO_2_ nanosphere was also served as a precursor in the process of TiO_2_ nanotube synthesized. The typical hydrothermal process in a certain concentration sodium hydroxide aqueous solution was applied to synthesize TiO_2_ nanotube (Kumar et al., 2016). At first, 2.5 g TiO_2_ sample was dispersed in 200 ml of NaOH solution (10 M). After stirring in the circumstance temperature for 1 h, the obtained slurry was transferred and sealed in a Teflon-lined autoclave for the hydrothermal treatment under 130°C in an atmospheric pressure for 20 h. The certain volume of 0.1 mol/L HCl and ethanol solution was applied to wash the precipitate alternately after the supernatant cooling down to the circumstance temperature. For the last step, the obtained solid was dried at 70°C under the atmosphere for 12 h. The hydrothermal process was also employed to prepare TiO_2_ nanoplate. In the beginning, 10 ml titanium tetra-isopropanolate was dissolved in 1.2 ml hydrofluoric acid with continuous stirring for 30 min. After that, a hydrothermal treatment was carried out for 24 h under 180°C and atmospheric pressure. The products were washed alternatively by water and ethanol in centrifugation until the final pH of the suspension reached 7. The sample was then dried for 12 h at 70°C. It should be noted that all TiO_2_ samples were calcined at 350°C under the atmosphere for 5 h in the last step of preparation.

After obtaining the TiO_2_ precursors with different morphologies, the corresponding Ag_2_O-TiO_2_ particles were prepared using a precipitation method (Zhou et al., 2010). TiO_2_ precursors (0.5 g) of each kind were dispersed in 100 ml of distilled water, followed by dissolving 0.725 g AgNO_3_ to each suspension while stirring (weight ratio of Ag_2_O:TiO_2_ = 1:1). Afterward, the excess amount 0.2 M NaOH solution was added to the mixture with continuously stirring to gain the precipitate. Finally, various Ag_2_O-TiO_2_ samples were obtained after washing and drying. Due to the control of the compositions, the morphologies of TiO_2_ were considered to be unchanged after compounding Ag_2_O. The obtained Ag_2_O-TiO_2_ nanosphere, Ag_2_O-TiO_2_ nanoplate, and Ag_2_O-TiO_2_ nanotubes were denoted as AS, AP, and AT, respectively.

### Characterization of Catalysts

The BET (Brunauer-Emmett-Teller) method was employed to detect the specific areas of the catalysts that were detected by N_2_ adsorption and desorption isotherms at 77 K with Micrometric Acusorb 2100E apparatus. In a typical procedure, the sample was disposed in vacuum to degassed prior to the measurement at 100°C for 1 h and then at 120°C for 2 h in turn. The crystal phase and structure of the samples were investigated using powder XRD (Shimadzu XRD-6000) for diffraction angle 2 h from 20° to 80° where a Cu target Kα-ray (operating at 40 kV and 30 mA, with *k* = 0.1541 nm). In the applied continuous mode, a nominal step interval of 0.0025° 2θ with a step time of 100 s was set. According to the diffraction peaks and the mean crystallite size was calculated by the Scherrer equation. Detailed morphologies and structures of the catalysts were observed under the HRTEM using JEM-2100. UV-vis absorption spectra of the samples were obtained by a UV-3600 plus (Shimadzu, Japan) apparatus. The particle sizes were analyzed at 25°C by dynamic light scattering (DLS) at a scattering angle of 173 with a Zeta sizer Nano ZS particle size analyzer (Beckman Coulter, Inc., USA).

### Colloidal Dispersion Stability Measurements

The dispersion stabilities of the nanoparticle suspensions were analyzed by the Turbiscan Lab® Expert type stability analyzer manufactured by Formulation (France) (Buron et al., 2004; Wiśniewska, 2010; Fang et al., 2012; Kang et al., 2012). A near-infrared light source λ = 880 nm based on multiple light scattering, transmission coefficients, and backscattered pulses was monitored by two simultaneous optical detectors. It should be noted that a fingerprint spectrum characterizing the dispersion performance of sample could be confirmed when the measurement frequency, scanning time, and scanning interval of the analyzer were set. The dispersion stability was evaluated by Turbiscan Stability Index (TSI) with the help of Turbiscan Easy Soft®. Based on the measured data, Turbiscan Stability Index (TSI) could be calculated using the following equation:

(1) $$\begin{array}{r} \text{TSI}=\frac{\sum_{\text{h}}\text{sca}{\text{n}}_{\text{i}}\left(\text{h}\right)-scan_{i-1}\left(\text{h}\right)}{\sum \text{h}} \end{array}$$

Where *h* is the height of the sample cell, and *scan*_*i*_(h) denotes the light transmission or backscattering obtained by the *i* th scan at height *h*. Larger TSI value indicates less stable the dispersed system.

Zeta potential profiles of the suspension system were measured using a zeta-potential measurement device (Delsa Nano C/SS). The specific operation process was used to prepare the suspension: 2-mg sample was dispersed into 20-ml solvents and ultrasonicated for 1 h.

### Photocatalytic Activity Measurements

The photoreforming H_2_ production experiments were carried out in a duplex Pyrex flask at nearly ambient temperature and −0.1 MPa pressure, where openings of the flask were sealed with a silicone rubber seals and glass lids. A 300W Xe arc lamp (50 W, 320–780 nm, Beijing Philae Technology Co., Ltd., China) was used as a light source and vertically placed at 10 cm away from the top of the photocatalytic reactor. The focused light intensity and area on the flask for xenon lamp were ca. 120 mW/cm^2^ and 0.2 cm^2^, respectively. In each photocatalytic experiment, 0.1 g total amount of catalyst (or mixed binary catalysts with different weight ratios) was suspended in 100-ml glycerol aqueous solution (containing 7 vol% of glycerol). Based on the previous study, different weight ratios were selected as: 20% of AS with 80% of AP (denoted as 1AS-4AP), 40% of AS with 60% of AP (denoted as 2AS-3AP), 60% of AS with 40% of AP (denoted as 3AS-2AP), 80% of AS with 20% of AP (denoted as 4AS-1AP). Before each test, the suspensions were stirred for 30 min and maintained in ultrasonic agitation for another 30 min to maintain the initial dispersion stability. Every effort was made to ensure that there was no external interference making sense to the colloidal stability during each experiment. The produced gaseous products were detected by gas chromatographer with a TCD detector (GC-2014c AT, Shimadzu, Japan) and 5Å molecular sieve column using N_2_ as a carrier gas. The following equation group would be applied to calculate the apparent quantum efficiency (AQE) and light-to-hydrogen energy conversion efficiency (LTH) according to the work by (Yu et al., 2011):

(2) $$\text{P}=\text{E}\times {\text{A}}_{\text{R}}$$

(3) $${\text{N}}_{\text{p}}^{\text{i}}=\frac{\text{pt}\bar{\lambda }}{\text{h }\cdot \text{ c}}$$

(4) $$\Phi_{\text{a}}=\frac{{\text{2R}}_{{\text{H}}_{2}}{\text{N}}_{\text{A}}\text{t}}{{\text{N}}_{\text{p}}^{\text{i}}}$$

(5) $$\eta =\frac{\Delta {\text{H}}_{\text{c}}^{0}{\text{R}}_{{\text{H}}_{2}}}{P}$$

Where *P* is radiation flux, *E* is average irradiance, *A*_*R*_ is light-receiving area of reactor; $N_{p}^{i}$ is number of incident photons, *t* is reaction time, $\bar{\lambda }$ is equivalent wavelength, *h* is Planck constant, *c* is constant speed of light;Φ_*a*_ is the apparent quantum efficiency (*AQE*) and R_H2_ is the obtained hydrogen production rate, *N*_*A*_ is the Avogadro constant; ηis defined as the light-to-hydrogen (*LTH*) energy conversion efficiency, and $\Delta {\text{H}}_{\text{c}}^{0}$ is the enthalpy of combustion of hydrogen.

### Density Function Theory Calculation

**Figure 1:**
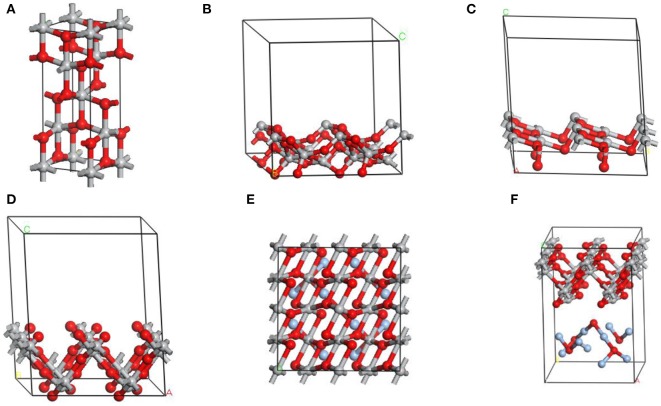
The image of **(A)** conventional cell of TiO_2_ in the anatase structure. Side view of crystal structures of different surface cells of TiO_2_ crystal used for surface energy calculation: **(B)** [101]; **(C)** [100]; **(D)** [001]. **(E,F)** Top and side views of the optimized geometric structures for Ag_2_O-TiO_2_ (001). Azury, red, and gray balls represent Ag, O, and Ti atoms, respectively.

Figures 1A–D show the conventional cell of anatase TiO_2_ and different surface cells introduced for surface energy calculation in this experience. As known, anatase TiO_2_ has a tetragonal structure [space group: I4_1_/amd, local symmetry: D_4h_ Long, 2013] that contains two titanium atoms and four oxygen atoms in its unit cell. A range of surface slabs could be created by optimizing bulk unit cell of anatase at its Miller indices by surface builder module in materials studio. In this work, we employed a flat slab with a thickness of 2 atomic layers, which was vertical to the surface and could extend indefinitely in the other two directions to simulate the surface of anatase TiO_2_ and call periodic boundary conditions. Besides, each repeated replica with a certain vacuum width of 12 Å constituted each surface cell in this work. For example, the proposed supercell model of anatase TiO_2_ (001) consisted of 16 titanium atoms, 32 oxygen atoms. The layer model of Ag_2_O coupled with anatase TiO_2_ (001) consisted of 16 titanium atoms, 37 oxygen atoms, and 10 silver atoms shown in Figures 1E,F. The atomic concentration (the number of atoms that can fit into a given volume) of silver was about 15.87% (atomic fraction), which was referenced from the sample used in the experimental section. All calculations were performed with the CASTEP using a total energy plane-wave pseudo-potential method) module in Material Studio 7.0 on the basis of density function theory (DFT) (Payne et al., 1992). The expanding wave functions of the valence electrons using a plane wave (PW) basis set within a specified energy cut-off of 300 eV. In additionally, we described the exchange correlation energy with the generalized functional approximation of the Perdew-Burke-Ernzerhof gradient(GGA-PBE) (Perdew et al., 1996) and the pseudo-potential representation was in the reciprocal space (Troullier and Martins, 1991).

In the calculation, the k-point mesh generated by Monkhorst-Pack scheme was set as 2 ×2 ×2 over Brillouim zone with a k-point spacing of 0.025Å^−1^. The Broyden-Fletcher-Goldfarb-Shanno (BFGS) method was set to relax the structure and the thresholds for the converged structure were set as following: energy change per atom was <2.0 ×10^−5^eV; residual force was <0.05 eV/Å; the displacement of atoms during the geometry optimization was <0.002 Å; and the residual bulk stress was <0.1 Gpa.

The thermodynamic stability of a given surface is dependent on its surface energy and a positive low value indicates a stable surface. The surface energy (Esurf) in a slab model could be calculated by (Meng et al., 2016):

(6) $$\begin{array}{r} {\text{E}}_{\text{surf}}=\left[{\text{E}}_{\text{slab}}-\left({\text{N}}_{\text{slab}}/{\text{N}}_{\text{bulk}}\right)E_{\text{bulk}}\right]/\left(2\text{A}\right) \end{array}$$

where E_slab_ and E_bulk_ represents the total energies of the surface slab and the bulk unit cell, respectively. N_slab_ and N_bulk_ are the numbers of atoms contained in the slab and the bulk unit cells, respectively, while A is denoted the unit area of the surface and “2” means that the flat slab has two faces along the z-axis. The surface energy was calculated by the CASTEP module in Materials Studio (MS) on the basis of DFT.

## Results and Discussion

### Photocatalyst Characterization

**Figure 2:**
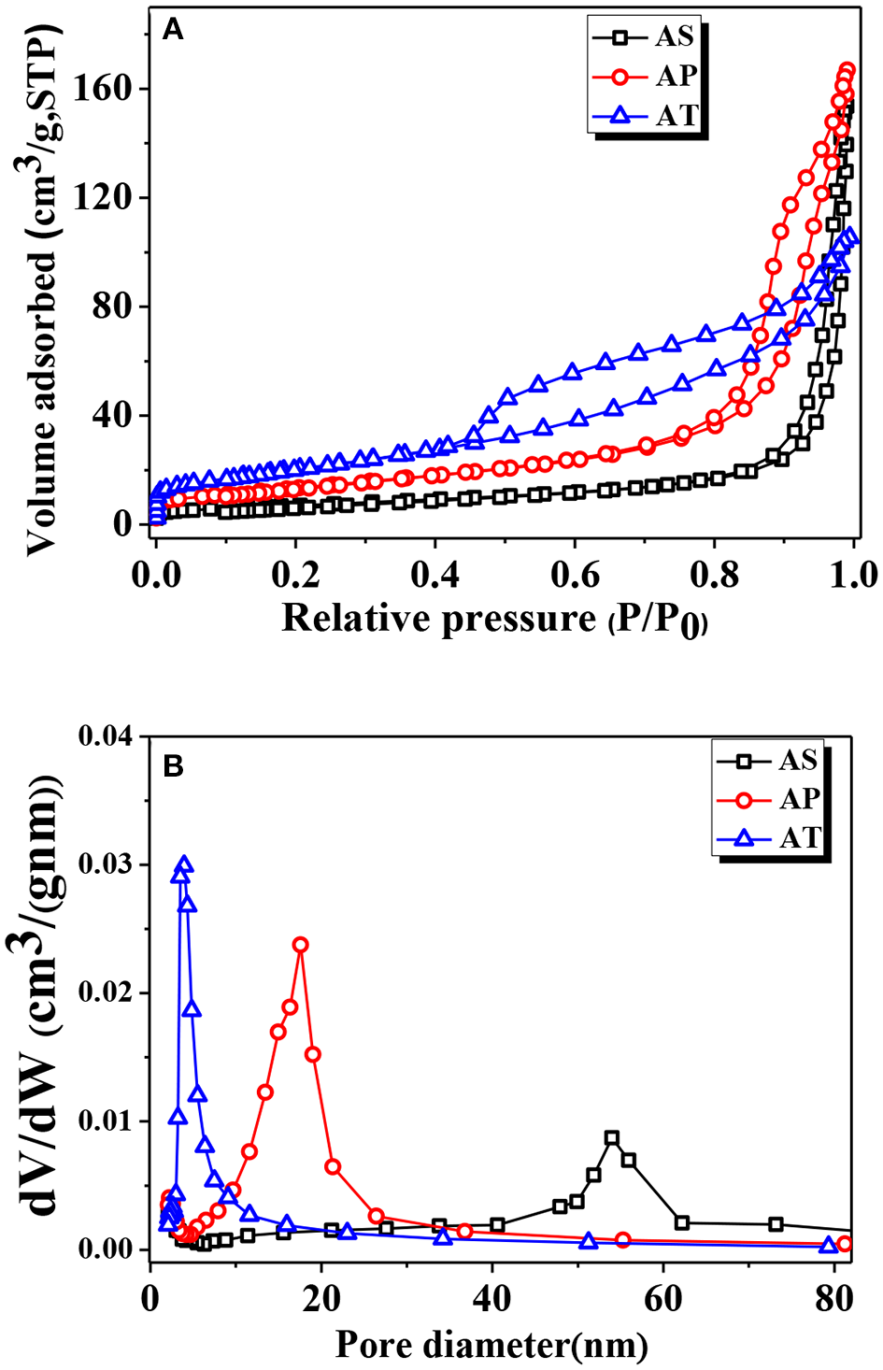
**(A)** Nitrogen adsorption–desorption isotherms and **(B)** pore size distributions [Barrett, Joyner, and Halenda (BJH) desorption] of Ag_2_O-TiO_2_ nanosphere (AS), Ag_2_O-TiO_2_ nanoplate (AP), and Ag_2_O-TiO_2_ nanotube (AT) samples.

The pore structures and BET surface areas of the as-prepared samples were detected by the N_2_ adsorption–desorption measurement. Figure 2 showed the isotherms and the corresponding pore size distribution curves of the samples. According to the International Union of Pure and Applied Chemistry (IUPAC) classification, type IV isotherm is the most approximate to the nitrogen adsorption–desorption isotherms of all samples, indicating the presence of mesoporous structure (2–50 nm). The shapes of hysteresis loops were of type H_3_ at the relative pressure value of range of 0.8–1.0, suggesting the presence of slit-like pores due to the stacking of TiO_2_-based particles. The Barrett, Joyner, and Halenda (BJH) method was used to obtain the pore size distribution curve from the desorption branch of the nitrogen isotherm. After calculating through the BJH method, the pore diameters for AS, AP, and AT were about 31.62, 16.58, 5.9 nm, and the BET surface areas of AS, AP, and AT were 25.31, 48.79, and 73.61 m^2^g^−1^; the related details were listed in Table 1.

**Table 1:** Physical properties of the prepared photocatalysts.

**Sample**	**S_**BET**_ (m^**2**^g^**−1**^)**	**Pore volume (cm^**3**^g^**−1**^)**	**Average pore size (nm)**	**Particle size (nm)^a^**	**Crystal size (nm)^b^**
AS	25.31	0.24	31.62	9.9	36.16
AP	48.79	0.26	16.58	10.95	33.67
AT	73.61	0.17	5.9	15.41	27.78

a *Ag_2_O particle sizes determined from transmission electron microscopy (TEM) profiles (statistics on 40 particles randomly picked up from images)*.

b *Ag_2_O crystal sizes determined from X-ray diffraction (XRD) patterns*.

**Figure 3:**
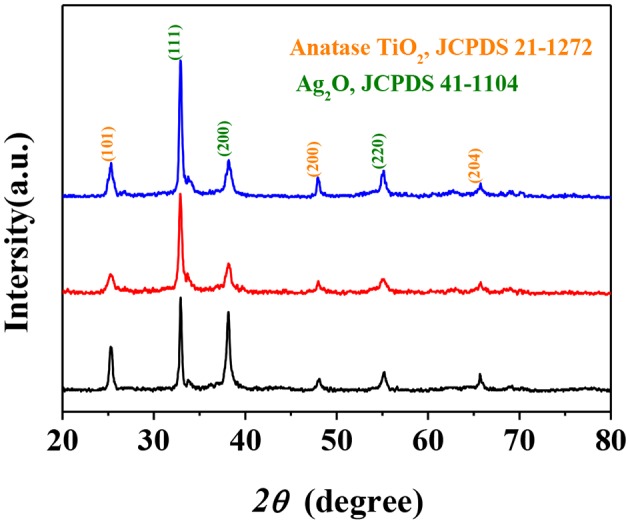
X-Ray Diffraction (XRD) patterns of Ag_2_O-TiO_2_ nanosphere (AS), Ag_2_O-TiO_2_ nanoplate (AP), and Ag_2_O-TiO_2_ nanotube (AT) samples.

The crystal structure and crystallinity of the synthesized photocatalysts were investigated using powder XRD analysis, and the results were demonstrated in Figure 3. The peaks at 2θ angles of 25.34, 48.08, and 64.52° for all samples corresponded to the (101), (200), and (204) crystal planes of anatase TiO_2_ (JCPDS 21-1272). Additionally, the diffraction peaks at 2θ angles of 32.9, 48.40, and 55.18° confirmed the presence of (111), (200), and (220) crystal planes of cubic Ag_2_O (JCPDS 41-1104). The appearance of Ag_2_O as a secondary phase in all samples indicated that Ag_2_O was well compounded with TiO_2_ particles of three different morphologies by the described synthesis method. As a matter of fact, such structures for all the samples may be beneficial for electron transfer which could be a benefit to the photocatalytic performance (Tan et al., 2003).

**Figure 4:**
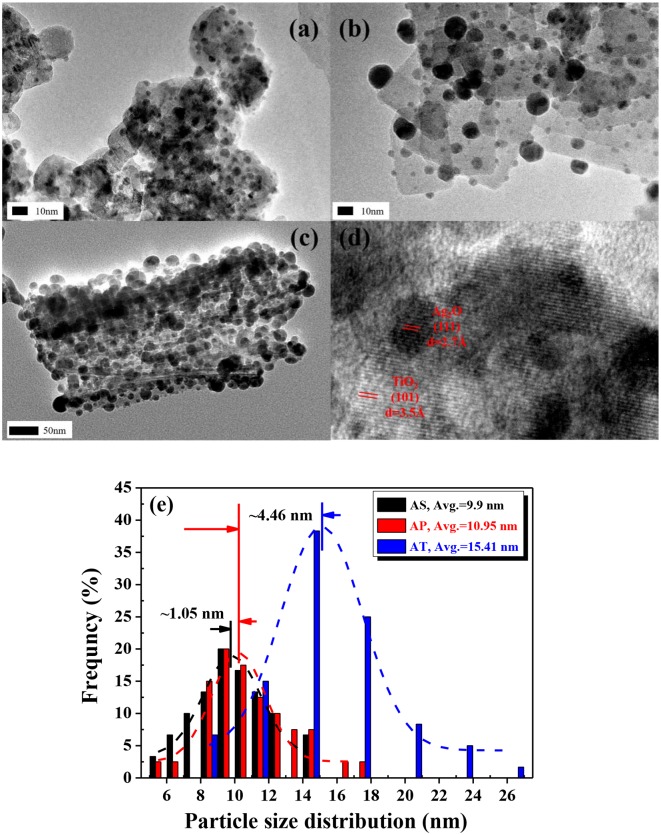
High-resolution transmission electron microscopy (HRTEM) images of: **(a)** Ag_2_O-TiO_2_ nanosphere (AS), **(b)** Ag_2_O-TiO_2_ nanoplate (AP), **(c)** Ag_2_O-TiO_2_ nanotube (AT), **(d)** the lattice of TiO_2_ and Ag_2_O, and **(e)** particle size distributions of Ag_2_O on AS, AP, and AT, respectively.

As seen in the HRTEM image (Figure 4a), Ag_2_O grains were regarded as much tinier compared to the bright part and could be identified as dark spots on the surface of the nearly transparent TiO_2_ nanospheres. The average particles size of Ag_2_O particles was calculated as 9.9 nm from respective HRTEM image (analyzed by Nano Measurer 1.2.0 software®). In Figures 4b,d, Ag_2_O particles were also tiny and well-dispersed in the way of anchoring tightly onto the surface of the TiO_2_ nanoplates. The Ag_2_O nanoparticles on TiO_2_ nanoplates are very stable and will not break even after ultrasonic treatment, which is meaningless. It could be observed that the loaded Ag_2_O particles had a fairly wide range of sizes that varied from 5.64 to 17.93 nm. The HRTEM image of Figure 4c revealed that the structure of the prepared TiO_2_ nanotubes was of cylindrical shape and hollow inside. The outer and inner diameters of the tube were about 7 and 4 nm, respectively. Similarly, the black spot on the surface of the AT in the HRTEM image implied to the presence of the Ag_2_O nanoparticles. In the previous observation of Ag_2_O-TiO_2_ photocatalyst, some of the Ag_2_O particles could be reduced to metallic Ag particles (Wang et al., 2017b). The average Ag_2_O size (20 measurement objects were randomly selected) over AT was 15.41 nm. Furthermore, almost no free Ag_2_O was found in the background of the HRTEM images, which could confirm a high loading rate of the Ag_2_O particles. The histogram in Figure 4e demonstrated the identified size distributions of Ag_2_O particles in each sample. According to the related work (Ren and Yang, 2017), the influence of the ununiform distribution of the Ag_2_O nanoparticles could be ignored regarding to the H_2_ yield. In fact, the photoelectrochemical properties of the catalysts synthesized with the same idea had been examined in our previous work which showed a competitive performance (Wang C. et al., 2019).

**Figure 5:**
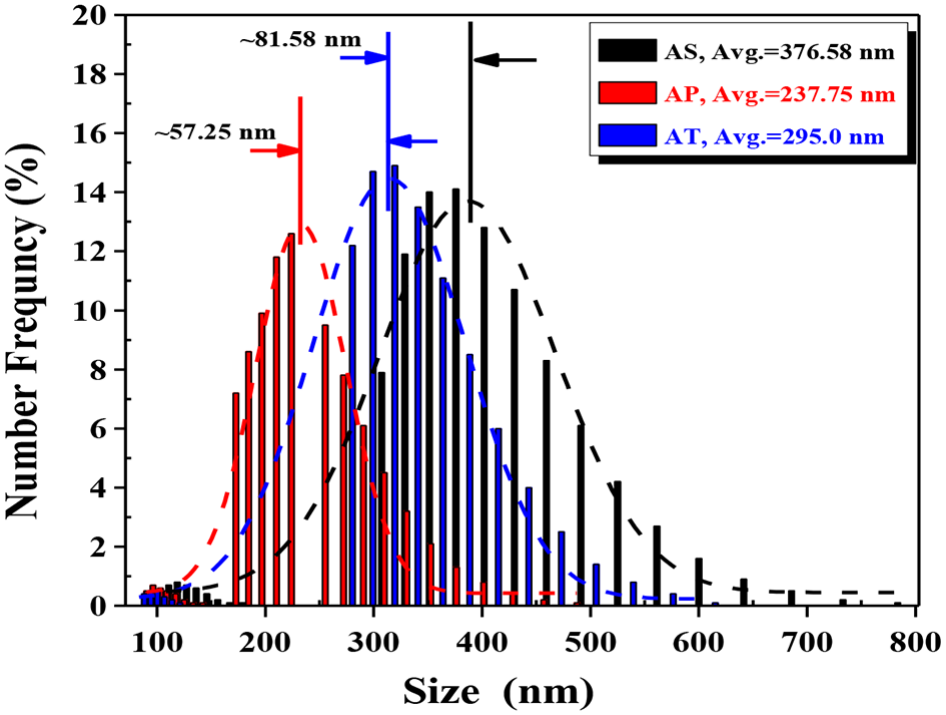
Particle size distribution histograms of dynamic light scattering (DLS) for the TiO_2_-based particles prepared. Average particle size is calculated from the Gaussian fittings of the histograms: Ag_2_O-TiO_2_ nanosphere (AS), Ag_2_O-TiO_2_ nanoplate (AP), and Ag_2_O-TiO_2_ nanotube (AT).

The apparent sizes of catalyst particles were measured in aqueous suspensions with certain concentrations using dynamic light scattering (DLS) technique. Just as the histogram in Figure 5 has shown, it could be observed that there was a wide range of particle diameters and particle aggregations. From the results of DLS measurements, two peaks were detected for all samples, about 5% of the detected particles for all those three samples were in the diameters between 90 and 150 nm. These parts might be formed by individual particles. The other parts of the detected particle sizes for AS, AP, and AT were in the range varying from 300 to 700 nm, 170 to 400 nm, and 250 to 550 nm, respectively, which might reflect their sizes of aggregations.

**Figure 6:**
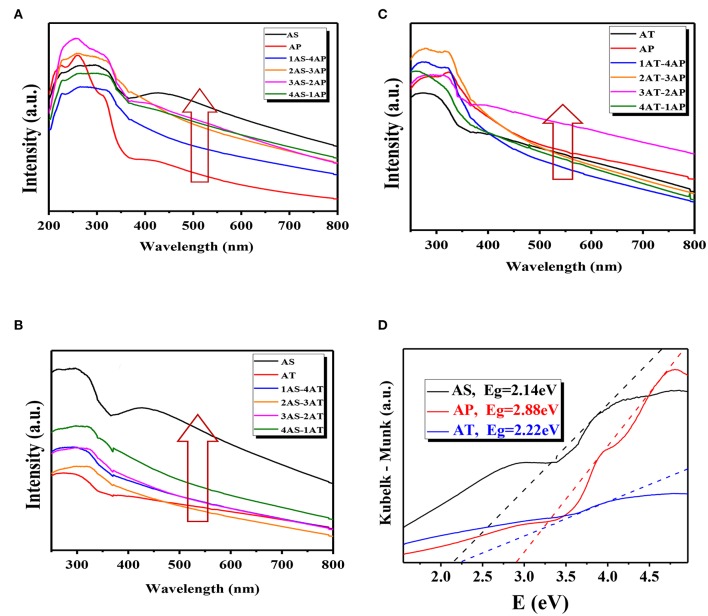
UV-vis absorption spectra **(A–C)** and the Kubelka–Munk diagrams (**D**, obtained from the data of the UV- vis absorption spectra) measured at different mixing binary component systems: **(A)** AS-AP, **(B)** AS-AT, and **(C)** AP-AT. AS, Ag_2_O-TiO_2_ nanosphere; AP, Ag_2_O-TiO_2_ nanoplate; AT, Ag_2_O-TiO_2_ nanotube.

Figure 6 showed the UV-visible absorption spectra for the binary mixing systems with the Kubelka–Munk diagram for apparent band gap energies (E_g_) to understand the optical properties and dispersion stability of the binary system, calculated by the Tauc equation in the following (Grover et al., 2013): (αhν)^n^ = hν-E_g_, where ν is frequency, h is Plank's constant, and *n* = 0.5 for indirect semiconductor, α is absorption coefficient, and E_g_ is the band gap energy. In fact, the absorptions above 400 nm in catalyst samples were ascribed to the presence of Ag_2_O as a functional visible-light sensitization compound which possessed both a tough and wide absorption band in the visible-light region (Zhou et al., 2010). The wavelength thresholds of the single-component system AS, AP, and AT were calculated to be 450, 450, and 520 nm, corresponding to the bandgaps of 2.75, 2.75, and 2.40 eV, respectively. The calculated E_g_ for AS, AP, and AT in this study were at the same level of the reported values of Ag_2_O-TiO_2_ (varied from 2.18 to 2.88 eV) (Zhou et al., 2010; Kumar et al., 2016; Ren and Yang, 2017). Here, it should be noted that 20% of AS and 80% of AP were denoted as 1AS-4AP. And other denote similar to the denoting rules. As known, the optical properties of TiO_2_ nanoparticles were sensitive to their morphologies, therefore the peak intensity differences of UV-vis absorption spectra for the single-component systems were mainly due to their morphological diversities. It could be deduced from the peak intensities of UV-vis light absorption (shown in Figure 6) that the light absorption abilities could be summarized as AS > AP > AT. The present result may be probably caused by the microscopic spatial structures of the materials and the different promoting effects of Ag_2_O for TiO_2_ with different morphologies. As shown, the light absorption capacity of AS-AT binary nanoparticle system was stronger than that of the AS single-component system. There was no obvious difference between AP and AT single-component systems in the light absorption capacities. Compared to AT or AP single-component systems, AT-AP binary system had not been improved in light absorption capacity. Generally speaking, the light absorption capacities of the binary systems located between the highest (AS) and lowest (AT) single-component system. From the results, the system of 60% AS mixed with 40% AP (3AS-2AP) exhibited the highest absorption capacity compared to other AS-AP binary systems, AS-AT systems, and AT-AP systems. Since the obtained specific surface areas and light absorption properties of Ag_2_O-TiO_2_ materials had no direct and obvious effect on the UV-vis absorption experimental results of the binary systems, it could be considered that the differences of light absorption properties might be caused by their different dispersion stabilities.

### Dispersion Stability Analysis

**Figure 7:**
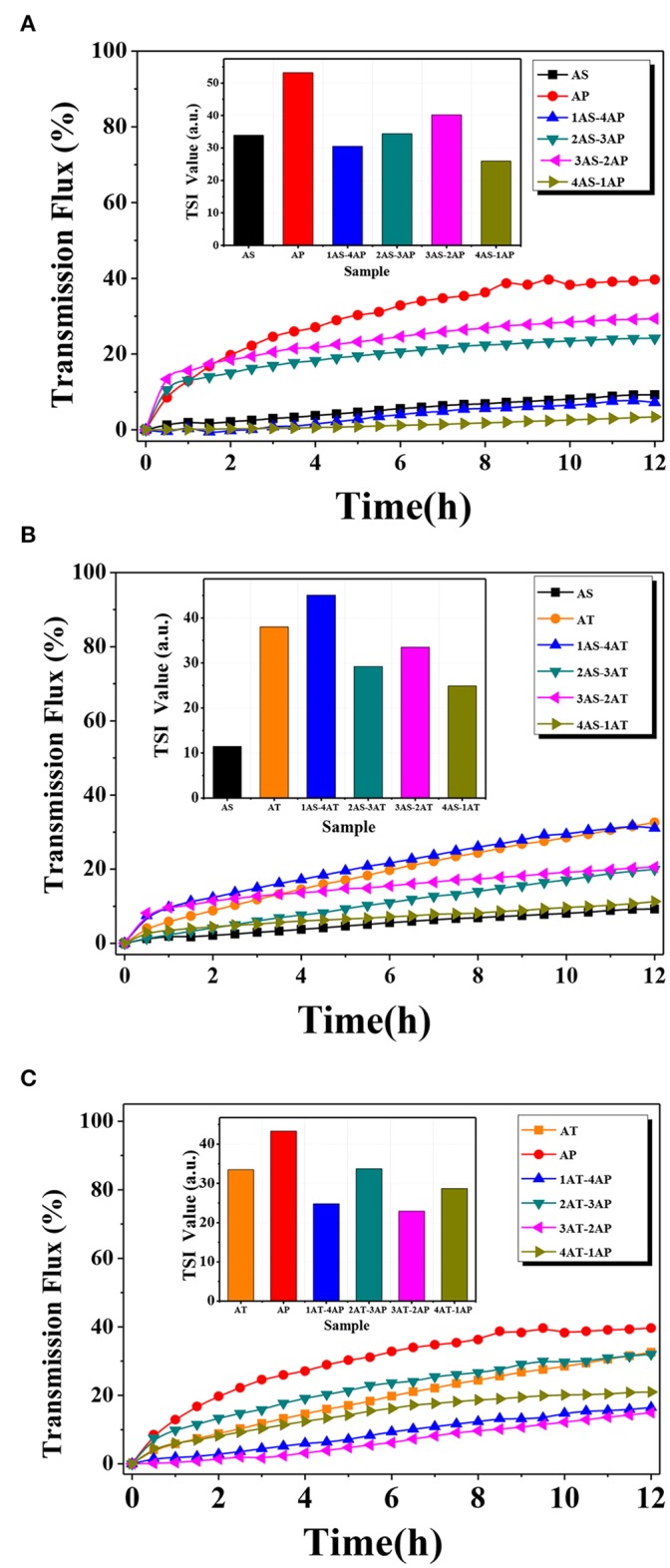
Transmission flux and the absolute value of Turbiscan Stability Indexes (inset) of the catalysts (total concentration of suspension = 0.1 wt.%) at different mixing binary component systems: **(A)** AS-AP, **(B)** AS-AT, and **(C)** AP-AT. AS, Ag_2_O-TiO_2_ nanosphere; AP, Ag_2_O-TiO_2_ nanoplate; AT, Ag_2_O-TiO_2_ nanotube.

**Figure 8:**
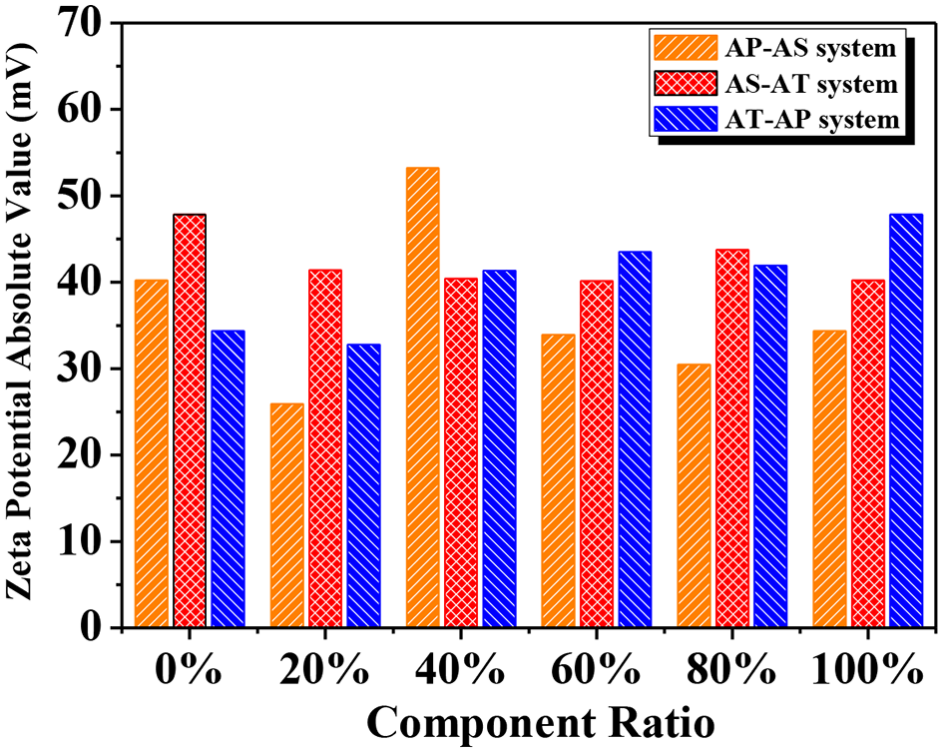
Zeta potential of different binary component systems (the x axis represents the mass ratio of the former component in the mixed system).

The dispersion stability could be analyzed by Turbiscan Stability Index (TSI), and the obtained TSI values for different systems were plotted in Figure 8. It should be noted that the TSI value shows a negative correlation to the dispersion stability for the suspension, and the increase of TSI value indicates a fast sedimentation process and a large thickness of the sediment. According to the results shown in Figure 7, AS single-component system performed the best dispersion stability among all samples, and the addition of AS with certain concentration improved the dispersion stability of AP- and AT-based systems, respectively. In the case of AP-AT binary system, the TSI values of the selected AT-AP binary systems were smaller than that of AT and AP single-component system, indicating the enhancing dispersion stabilities. Such increase in dispersion stability was possibly related to a comprehensive effect of electrostatic repulsion and steric hindrance according to the DLVO theory (Liu et al., 2015a). Generally speaking, this effect was caused by the reduction of particle collision frequency and agglomeration tendency.

The results of average transmission flux were also depicted in Figure 7. There was a fact that particles being homogeneously dispersed in water would block most of the laser to the detector, resulting in low transmission flux, while the formed sedimentations could not block most of the upper laser, so that the total transmission flux would be high. As the time increased, the transmission flux increased from 0 to 42% for the single-component systems, meaning agglomeration and sedimentation of AT and AP particles occurred due to the van der Waals force and the gravitational force. In Figure 7A, the dispersion stability of AP single-component system was significantly improved by mixing AS (AS varied from 0 to 40% of the mixing particles, while the mass of the mixing particles was 0.1 wt.% of total mass of the suspension). Nevertheless, the excessive AS (60%) declined dispersion stability of the suspension. It could be inferred that the depletion interaction between AS and AP was emphasized when reaching a critical ratio, and such depletion interaction between particles with different shapes might influence the dispersion stability (Mason, 2002; Zhang et al., 2013). In the case of AS-AT systems (Figure 7B), AT showed worse dispersion stability than AS with the same total concentration. This result suggested that AT in the suspension was easier to agglomerate resulting in the formation of large AT particles. Unfortunately, there was no obvious improvement in dispersion stability even mixing AS to AT. In Figure 7C, the dispersion stability of AT-AP binary system had been improved compared to AT or AP single-component system, indicating the generation of a strong electrostatic repulsion between the AT and AP solid surface.

Zeta potential (ζ) is widely recognized as an indicator of the stability of colloidal dispersions, revealing the potential difference between the dispersion medium and the fluidic connection of the stationary layer to the dispersed particles. Figure 8 displayed the absolute values of ζ for the single-component systems and the binary component systems. According to the previous study, the obtained values were large enough to maintain a relatively high stability (Patel and Agrawal, 2011). According to the most widely accepted DLVO theory, colloid stability depends on the sum of van der Waals attractions and electrostatic repulsive forces (Missana and Adell, 2000). The ζ value could provide information of the electrostatic repulsive force. On the other hand, the van der Waals force relies on the Hamaker constant, and this constant is determined by particle spatial configurations and other properties without considering the influence of an intervening medium between the two particles of interaction. When the Hamaker constant is small, the reflecting van der Waals force is weak, the low electrostatic repulsion reflected by small ζ may be appropriate to ensure colloid stability (Kim et al., 2014). Therefore, it is common to come across stable colloids with low ζ values.

### Photoreforming Hydrogen Production

**Figure 9:**
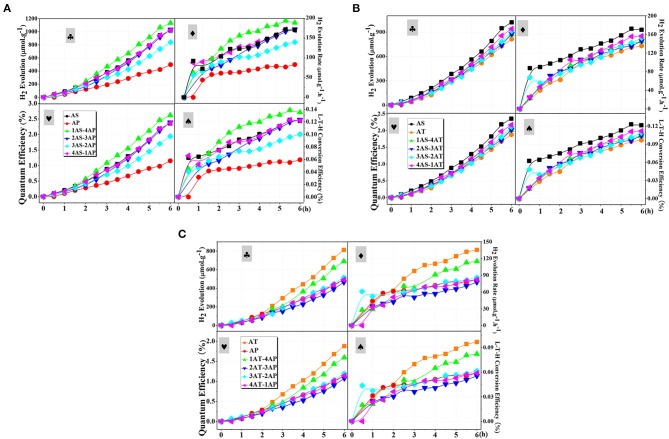
Typical time course of: 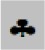 H_2_ production amount, 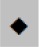 hydrogen evolution rate, 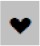 quantum efficiencies, and 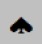 light-to-hydrogen energy efficiencies over the as-prepared photocatalysts in 7 vol.% aqueous solution of glycerol for different mixing binary component systems: **(A)** AS-AP, **(B)** AS-AT, and **(C)** AP-AT. AS, Ag_2_O-TiO_2_ nanosphere; AP, Ag_2_O-TiO_2_ nanoplate; AT, Ag_2_O-TiO_2_ nanotube.

The photoreforming H_2_ evolution over the single and binary systems were carried out in a glycerol-water system stimulated by 300 W xenon lamp. Figure 9 demonstrated the photocatalytic H_2_ evolution over time for different catalyst systems. As observed, the hydrogen production amount could be summarized as AP < AT < AS for the single-catalyst systems. Although AP with a large specific surface area and a high pore volume was considered as a two-dimensional (2D) material for excellent photo-generated carrier transfer property (Amano et al., 2009), the AP system did not exhibit a competitive photocatalytic hydrogen production performance. According to the analysis dispersion stability, both zeta potential and TSI value showed the poor dispersion stability of AP. In other words, the dispersion stability had a strong influence on hydrogen production performance. To be noted, AT with a 1D nanostructure exhibited higher photocatalytic activity among the single-component systems due to a better delocalization effect of the excited photo-generated electron–hole pairs and well-developed space charge region that reduced the recombination of photo-generated charge species effectively (Toledo Antonio et al., 2010; Kim et al., 2012; Zhao et al., 2014). Still, these advantages may be largely neutralized by the poor dispersion stability of AT. The poor dispersion stability of AT can be confirmed from the TSI value and the transmission flux. This result further indicated the importance of dispersion stability for photocatalytic hydrogen production. Compared with AP and AT single-component systems, AS with the lowest surface area, exhibited the best photocatalytic hydrogen production performance.

Experiments were carried out to further confirm the role of binary systems with a high dispersion stability for photocatalytic hydrogen production. For AS-AP binary component system (Figure 9A), the photocatalytic activity of AP was significantly improved by mixing AS. From the results of the AS-AP binary systems, 3AS-2AP showed the least photocatalytic hydrogen production amount, while its dispersion stability appeared to be the worst of the binary systems. Since there were differences of catalytic performance and the dispersion stability between AS and AP, the binary system might present its catalytic performance within the range of those two single-component systems. Due to the doping of Ag_2_O–TiO_2_, a structure might be formed to display the antenna mechanism for promoting the catalytic activity, and the binary component system may have a more positive impact on the role of this mechanism compared to that of the single-component system (Wang et al., 2006). Among those binary systems, 20% AS and 80% AP system displayed the largest photocatalytic H_2_ production amount of 1,133.21 μmolg^−1^. In the suspension of AS-AT binary component system, the depletion interaction between AS and AT was weak, leading to little effect on the enhancement of dispersion stability. As illustrated in Figure 7B, the dispersion stability of AS-AT binary component systems was not effectively improved compared with AS and AT single-component suspensions; therefore, the photocatalytic hydrogen production performance was not enhanced (Figure 9B). In Figure 9C, the photocatalytic performance of the AT-AP binary component system was not significantly improved compared to that of single-component systems regardless of time effect on reaction kinetics, this result was highly consistent with the previous result of bare TiO_2_ catalyst (Cai et al., 2018). The dispersion stability results of AT-AP binary systems were roughly consistent with the trend of photocatalytic activities. If not considering AT single-component system, 1AT-4AP binary system displayed the photocatalytic H_2_ production amount of 690.16 μmol·g^−1^ and was about 1.5 times higher than that of the AP single-component system. However, AT with complex spatial structure and electronic transmission characteristics may have special microscopic particle interaction forces, and this result was similar to that of our previous study of bare TiO_2_ nanotubes meaning that doping Ag_2_O did not significantly change the spatial interaction of TiO_2_ nanotube particles (Cai et al., 2018).

The light source at the range of 320–780 nm was used in this experiment. In the range of UV light irradiation, both TiO_2_ and Ag_2_O could be excited to generate the photogenerated electron-hole pairs, while the visible light irradiation is only absorbed by Ag_2_O, according to (R1) and (R2). The Ag_2_O would be *in situ* reduced by the electrons to Ag according to (R3). Then, the photo-generated holes on both TiO_2_ and Ag_2_O will produce reactive oxygen species ·OH. At the same time, according to (3) and (5), the O_2_ obtained by the Ag_2_O reduced would react with the electrons to more ·OH, which could improve the TiO_2_ photocatalytic activity (Ran et al., 2019; Chen et al., 2020). Because of the band gap of the Ag_2_O and TiO_2_, the Ag_2_O can be excited to produce h^+^ and e^−^ under the visible light and the electrons on the conduction would be transferred to the conduction band of TiO_2_ to produce H_2_. Thus, in this biphasic photocatalyst, the Ag_2_O acts as a visible light sensitizer to absorb more energy from the light source, while the Ag acts as an electron to transfer the photo-generated electrons to improve the H_2_ yield (Sadanandam et al., 2017).

$$\begin{array}{l} {\text{TiO}}_{2}\overset{\text{irradiation}}{\to }{\text{h}}^{+}+{\text{e}}^{-}\;(\text{R}1)\\ {\text{Ag}}_{\text{2}}\text{O}\overset{\text{irradiation}}{\to }{\text{h}}^{+}{\text{+e}}^{-}\;(\text{R2})\\ {\text{Ag}}_{2}\text{O}+{\text{e}}^{-}\to \text{Ag}+{\text{O}}_{2}\;(\text{R3})\\ {\text{h}}^{+}+{\text{H}}_{2}\text{O}\to •\text{OH}+{\text{H}}^{+}\;(\text{R4})\\ {\text{e}}^{-}+{\text{H}}_{2}\text{O}+{\text{O}}_{2}\to •\text{OH}+{\text{OH}}^{-}\;(\text{R5}) \end{array}$$

### Kinetic Analysis

**Figure 10:**
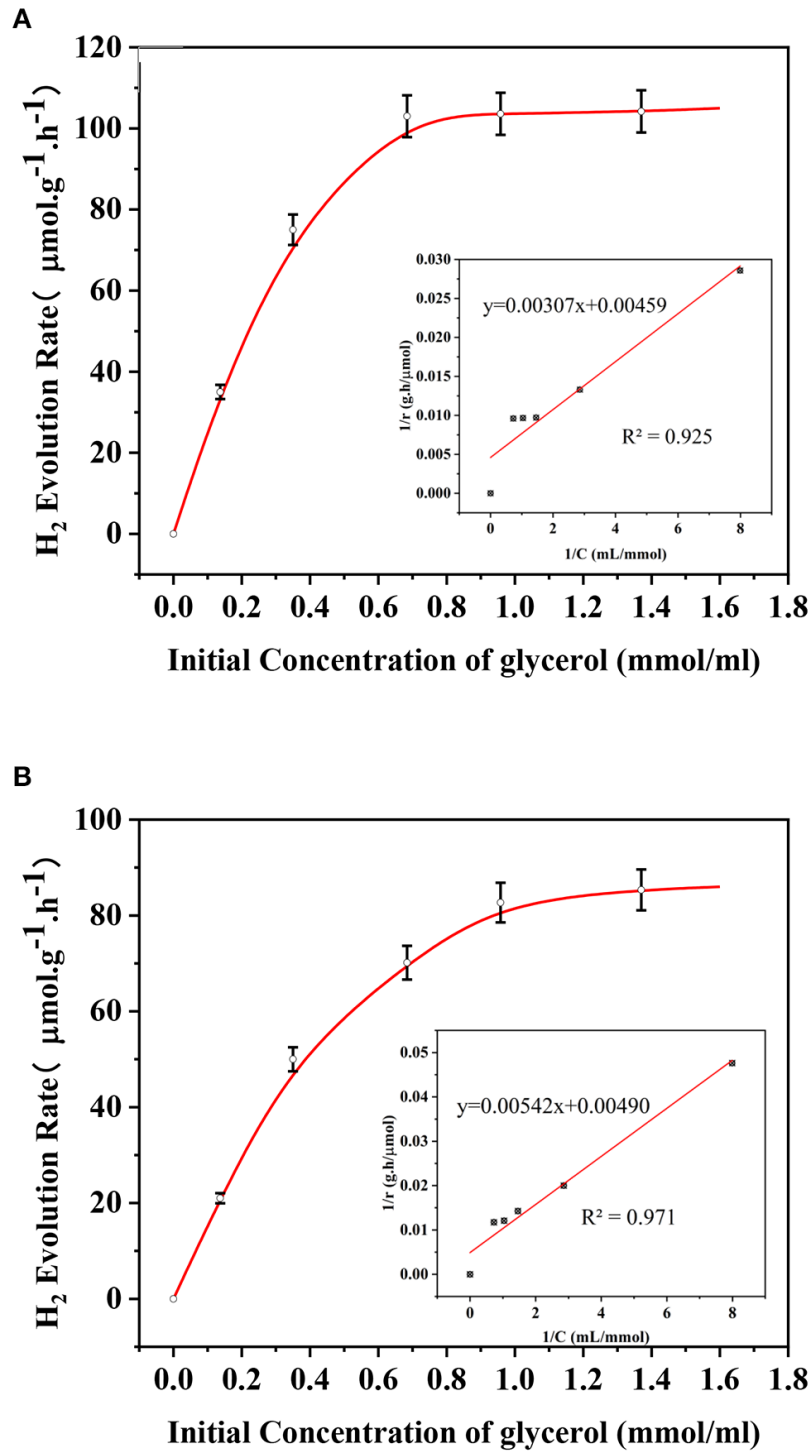
Initial H_2_ evolution rate curve **(A)** with stirring **(B)** without stirring and the fitting plot of 1/r vs. 1/C in the inset.

During a typical heterogeneous photocatalytic hydrogen production from photoreforming, the organic substrates are considered to be strongly adsorbed on the catalyst surface to promote the direct reaction between positive holes and organics rather than those in the solutions (Clarizia et al., 2017). The reaction rate could be described by Langmuir–Hinshelwood (L-H) kinetics, which is dominated by different rate-determining steps under different concentrations of the adsorbed species (Rivero et al., 2019). In fact, the initial concentration of the glycerol solution in this study is an effective means of reflecting kinetic behavior for hydrogen production (shown in Figure 10). For each experiment, 1-h irradiation without stirring was carried out for photocatalyst with a total mass of 0.1 g (80% of AP and 20% of AS which was proved to have a better self-dispersion stability among binary systems). Meanwhile, another set of experiment was conducted under the same condition except for the continuous stirring. The L-H kinetic model could be described as follows:

(7) $$\begin{array}{r} r_{a}=k_{a}\text{C}\left(1-\theta \right) \end{array}$$

(8) $$\begin{array}{r} r_{d}=k_{d}\text{C}\cdot \theta \end{array}$$

Assuming the equilibrium of absorption and desorption:

(9) $$\begin{array}{r} k_{a}\text{C}\left(1-\theta \right)=k_{d}\text{C}\cdot \theta \end{array}$$

(10) $$\theta =\frac{\frac{k_{a}}{k_{d}}\text{C}}{1+\frac{k_{a}}{k_{d}}\text{C}}$$

(11) $$r_{H_{2}}=k_{H_{2}}\cdot \theta =k_{H_{2}}\frac{\frac{k_{a}}{k_{d}}\text{C}}{1+\frac{k_{a}}{k_{d}}\text{C}}$$

(12) $$\begin{array}{r} \frac{1}{r_{H_{2}}}=\frac{1}{k_{H_{2}}}+\left(\frac{1}{\frac{k_{a}}{k_{d}}k_{H_{2}}}\right)\frac{1}{\text{C}} \end{array}$$

where *r*_*H*_2__ refers to H_2_ generation rate, the result of $\frac{k_{a}}{k_{d}}$ is the absorption equilibrium constant, *k*_*H*_2__ is the rate constant of hydrogen production, θ is the cover degree, and C is the initial concentration of glycerol solution. As shown, the results of the kinetic calculations fitted well with the experimental data for both cases, indicating the wide applicability of *L-H* kinetics for photoreforming reactions. The increase of reaction rates was significantly reduced when the initial concentrations of the glycerol solutions came to 1 mmol/ml for both cases reflecting the key concentration of the speed-limiting step switching. As obtained, the values of *k*_*H*_2__ and $\frac{k_{a}}{k_{d}}$ of the case under stirring were 217.9 μmol/g·h and 1.39 ml·μmol^−1^, respectively. On the other hand, the corresponding values of *k*_*H*_2__ and b were 204.08 μmol/g·h and 0.90 ml·μmol^−1^ for the case of colloidal system with a self-dispersion stability. From these results, different reaction rate constants and absorption equilibrium constants for the two cases denoted their different mass transfer characteristics when using the same type of catalyst combination under the same light intensity and temperature. Although the binary system with a better self-dispersion stability was still less efficient than the mass transfer promoted by continuous stirring, it still can be kept at a high level of pseudo first-order kinetic constants. It is worth noting that the difference of hydrogen production rate constants between stirring and self-dispersion stability was <6%, showing that the mass transfer efficiency of the binary system for hydrogen production was high within the selected concentration range.

### Density Function Theory Study

The crystal lattice of Ag_2_O and bulk anatase TiO_2_ had been optimized before calculated Ag_2_O and TiO_2_ surface and the lattice constant of Ag_2_O after optimizing is 4.728Å. The lattice parameter of bulk anatase TiO_2_ (a = 3.776Å, b = 3.776Å, c = 9.486Å), calculated by DFT method, corresponded to the experimental data (Arlt et al., 2000). The equilibrium morphology of a crystal was determined by its surface energy and the related growth rate of various surfaces (Cooper and de Leeuw, 2003), which means the certain surface with a high surface energy was supposed to have a great growth rate, and these fast growing surface would not be presented in the resulting crystal morphology. On the contrary, those surfaces with low surface energies and hence slow growing rates had the opposite situation in the resulting crystal morphology (Gao et al., 2013). According to the surface energies theory, the thermodynamic penalty for cleaving a surface from a bulk material was also detected. The calculated surface energies of three types of anatase TiO_2_ surfaces are shown in Table 2. According to Table 2, the surface energies of different anatase TiO_2_ surfaces followed the order of [001] > [100] > [101]. (101) surface with the lowest surface energy was the main cleavage and was expressed planes in the equilibrium morphology of anatase TiO_2_ crystal which matched well with the XRD results (in Figure 3). The total energy of a unit cell with formula Ti_2_O_4_ of anatase crystal is shown in the footnote in Table 2.

**Table 2:** Total energies of surface cells and surface energies of different surfaces of anatase TiO_2_.

**Crystal surface**	**a/******	**b/******	**c/******	**Total energy/eV**	**Cell formula**	**Surface energy/(J·m^**−2**^)**
(101)	7.6441	10.9146	11.2636	−19837.6970	Ti_8_O_16_	0.74
(100)	7.5684	10.9164	10.7824	−19838.61	Ti_8_O_16_	0.78
(001)	10.7033	10.9164	12.0138	−39671.58	Ti_16_O_32_	1.53

*The total energy of a unit cell with formula Ti_2_O_4_ of anatase crystal is −4961.973 4eV*.

**Figure 11:**
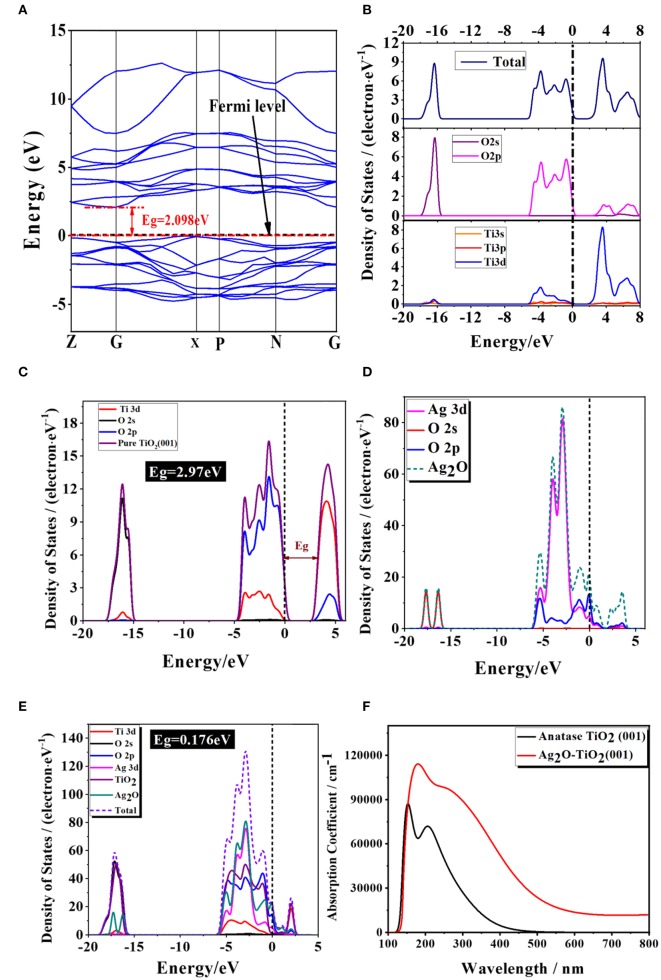
The image of **(A)** calculated properties of pure anatase TiO_2_ band structure; **(B)** the density of states; the density of states on Ag, Ti, and O atoms for **(C)** anatase TiO_2_ (001) slab, **(D)** Ag_2_O and **(E)** Ag_2_O-TiO_2_ (001), and **(F)** the calculated light absorption of the pure anatase TiO_2_ and Ag_2_O-TiO_2_. The short dotted lines denote the Fermi level.

Figures 11A,B displayed the obtained band structure and density of states DOS [including the total density of states (TDOS) and the partial density of states (PDOS)] of pure anatase TiO_2_. The calculated band gap of pure anatase TiO_2_ was 2.098 eV, which was underestimated comparing to the experimental E_g_ = 3.2eV for the reason that the framework of the DFT would not take discontinuity of the exchange-correlation potential into account (Stampfl and Van de Walle, 1999; Zhao et al., 2015). The valence band of the pure anatase TiO_2_ phase was mainly composed of the part from −20 to −15 eV and the one from −5 to 0 eV. The former part was mainly consisted by the O_2s_ states, which were far away from the top of the valence band, and other electronic states were not obvious, so it had little impact on the physical properties of objects. The latter part was mainly consisted of both the O_2p_ states and Ti3d states. As the Ti_3d_ states were split into two parts (the t_2g_ and e_g_ states) in an octahedral ligand field with O_h_ symmetry, the CB could be divided into the lower and the upper parts (Fang et al., 2014). Additionally, the PDOS diagram showed that the conduction band mainly consisted by the Ti_3d_ states. In general, the Ti_3d_ states act a dominate role in the conduction band in the pure TiO_2_, while the O_2p_ states act in the valence band. This result implies that the main cause of optical absorption is electrons transiting from O_2p_ to Ti_3d_ states, which is corresponding to the previous theoretical researches (Cao et al., 2014; Wang et al., 2017a).

As compared to the 2.97V of E_g_ of pristine TiO_2_ (001), the E_g_ was found to narrow to 0.176eV for Ag_2_O coupled with TiO_2_ (001). In general, in pure TiO_2_, the Ti_3d_ states act a dominate role in the conduction band, while the O_2p_ states act in the valence band. This result implies that the main cause of optical absorption is electrons transiting from O_2p_ to Ti_3d_ states, which is corresponding to the previous theoretical researches (Melrose and Stoneham, 1977; Wang et al., 2017a). Our previous studies reported that incorporation of Ag_2_O into TiO_2_ can extend the spectral response to the visible-light region, and the photocatalytic activity is greatly enhanced in hydrogen production from glycerol:water mixture systems (Melrose and Stoneham, 1977; Wang et al., 2017b). Our calculated results were well-agreed with these experimental results. The projected density of states has been corrected to the partial density of states (PDOS) were calculated and plotted in Figures 11D–F in order to further gain the origin of electronic structures of Ag_2_O-coupled TiO_2_. For comparison, the DOS and PDOS of pure TiO_2_ and Ag_2_O were also displayed in Figures 11D,E, respectively. According to the calculated results, the top of the valance band of pure Ag_2_O consists mainly of Ag_3d_ states, while the bottom of the conduction band is dominated by O_2_p states. Whenever coupled on perfect and deficient TiO_2_ (001) surfaces, the characteristic DOS of Ag_2_O accounted for the fact that Ag_2_O is preserved well. Additionally, calculations indicated that the shift of Fermi level is up-shifted by 1.1 eV relative to the position of conduction band of TiO_2_. The appearance of the down-shifted of the bottom conduction band indicated the appearance of the Ag_3d_ and O_2_p of Ag_2_O compared with pure TiO_2_ (001). For Ag_2_O-coupled TiO_2_, the splitting of the Ag_3d_ and O_2_p orbitals into occupied and unoccupied states will cause an impurity band in the forbidden gap, which would express as a weak but visible peak in the vicinity of the Fermi level in the image (Figure 11E). These effects may result in the band gaps being narrowed (Fang et al., 2014).

To further analyze the optical absorption spectrum of pure and coupled TiO_2_, we calculated the complex dielectric function ε(ω) = ε_1_(ω)−*iε*_2_(ω) according to the obtained electronic structures. Generally speaking, the imaginary part, ε_2_(ω), of the dielectric function could be evaluated from the momentum matrix elements between the occupied and unoccupied wave functions. ε_1_(ω), the real part of the dielectric function, could be evaluated from ε_2_(ω) by the Kramer–Kronig relationship (Sun and Wang, 2005). The absorption spectra were calculated based on the equation (Zhang et al., 2017):

(13) $$\begin{array}{r} \text{I}\left(\omega \right)=\sqrt{2}\omega {\left[\sqrt{\varepsilon_{1}^{2}\left(\omega \right)+\varepsilon_{2}^{2}\left(\omega \right)}-\varepsilon_{1}\left(\omega \right)\right]}^{1/2} \end{array}$$

In this equation, *I* represents the optical absorption coefficient, ω represents the angular frequency. Based on the calculated electronic structures, the optical absorption spectra of the pure TiO_2_ and Ag_2_O-coupled TiO_2_ were calculated and shown in Figure 11C. It could be very clearly observed that the pure TiO_2_ had nearly no response to the range of the visible range and only absorbed actively to UV light. In contrast, for Ag_2_O-coupled Ag_2_O, the narrowed band gap will effectively absorb light in visible range due to the formation of the localized mad gap level above the conduction band by Ag_2_O compounding. The result is in line with that of DOS.

## Conclusion

A series of Ag_2_O-TiO_2_ nanoparticles with different morphologies were prepared, and their dispersion stabilities in aqueous phase were investigated individually. Among those Ag_2_O-TiO_2_ composite catalysts, Ag_2_O-TiO_2_ nanosphere displayed a better colloidal dispersion stability in the suspensions. Using the as-prepared Ag_2_O-TiO_2_ catalysts, photoreforming H_2_ production was carried out from glycerol aqueous solution and the colloidal dispersion stability was found to be one of the dominant factors for heterogeneous catalysis in aqueous phase. Novel Ag_2_O-TiO_2_ binary component systems with proper ratios of mixture were successfully introduced to enhance the dispersion stability, thereby improving hydrogen production performance. Among the binary component systems, 20% Ag_2_O-TiO_2_ nanospheres mixing with 80% Ag_2_O-TiO_2_ nanoplates displayed the best photocatalytic activity with the maximum H_2_ production amounts around 11,33.21 μmolg^−1^. It was interesting that the difference of hydrogen production rate constants between continuous stirring and the binary system was <6%, indicating an efficient mass transfer of the binary system toward photoreforming hydrogen production. In order to further explore the mechanism, the photoelectrochemical characteristics are suggested for study in the future work. The proposed method of mixing Ag_2_O-TiO_2_ catalyst particles with different shapes could provide some inspiration to a more energy-efficient heterogeneous catalytic hydrogen production process.

## Data Availability Statement

The raw data supporting the conclusions of this article will be made available by the authors, without undue reservation, to any qualified researcher.

## Author Contributions

ZY and CW contributed experiment methods and design of DFT calculations. WZ and SM contributed the synthesis of the samples and the characteristics of the sample. YC, JZ, RS, and QS organized the literature research of this issue and wrote part of the manuscript. All authors contributed to manuscript revision, read, and approved the submitted version.

### Conflict of Interest

The authors declare that the research was conducted in the absence of any commercial or financial relationships that could be construed as a potential conflict of interest.
